# Isolation of single circulating trophoblasts from maternal circulation for noninvasive fetal copy number variant profiling

**DOI:** 10.1002/pd.6275

**Published:** 2022-12-08

**Authors:** Anna Doffini, Claudio Forcato, Chiara Mangano, Debora Lattuada, Roberta Aversa, Chiara Maranta, Emilia D. Giovannone, Genny Buson, Chiara Bolognesi, Rebecca Maiocchi, Martina Dori, Liyana Jamal, Raidah B. Ahmad, George S. H. Yeo, Tai Wai Yeo, Silvia Saragozza, Rosamaria Silipigni, Marta Serafini, Andrea Biondi, Sofia Perego, Patrizia Vergani, Enrico Ferrazzi, Paola Ricciardi‐Castagnoli, Thomas J. Musci, Francesca Romana Grati

**Affiliations:** ^1^ A. Menarini Biomarkers Singapore Pte Ltd, R&D department Singapore Singapore; ^2^ Department of Woman Child and Neonate, Obstetrics Unit, Fondazione IRCCS Ca’ Granda Ospedale Maggiore Policlinico Milan Italy; ^3^ Department of Maternal Fetal Medicine KK Women's and Children's Hospital Singapore Singapore; ^4^ Research and Development, Cytogenetics and Molecular Genetics TOMA Advanced Biomedical Assays S.p.A., Impact Lab Busto Arsizio Italy; ^5^ Laboratory of Medical Genetics, Fondazione IRCCS Ca’ Granda Ospedale Maggiore Policlinico Milan Italy; ^6^ Centro Ricerca M. Tettamanti, Department of Pediatrics University of Milano‐Bicocca Monza Italy; ^7^ Department of Obstetrics and Gynecology MBBM Foundation Onlus at San Gerardo Hospital Monza Italy

## Abstract

**Objective:**

To develop a multi‐step workflow for the isolation of circulating extravillous trophoblasts (cEVTs) by describing the key steps enabling a semi‐automated process, including a proprietary algorithm for fetal cell origin genetic confirmation and copy number variant (CNV) detection.

**Methods:**

Determination of the limit of detection (LoD) for submicroscopic CNV was performed by serial experiments with genomic DNA and single cells from Coriell cell line biobank with known imbalances of different sizes. A pregnancy population of 372 women was prospectively enrolled and blindly analyzed to evaluate the current workflow.

**Results:**

An LoD of 800 Kb was demonstrated with Coriell cell lines. This level of resolution was confirmed in the clinical cohort with the identification of a pathogenic CNV of 800 Kb, also detected by chromosomal microarray. The mean number of recovered cEVTs was 3.5 cells per sample with a significant reverse linear trend between gestational age and cEVT recovery rate and number of recovered cEVTs. In twin pregnanices, evaluation of zygosity, fetal sex and copy number profiling was performed in each individual cell.

**Conclusion:**

Our semi‐automated methodology for the isolation and single‐cell analysis of cEVTS supports the feasibility of a cell‐based noninvasive prenatal test for fetal genomic profiling.

## Key points

**What's already known about this topic?**

- Fetal circulating extravillous trophoblasts (cEVTs) have been isolated from maternal blood for both aneuploidy and copy number variant (CNV) classification.
- Previous reported methodologies require mostly manual low throughput workflows and identify putative fetal cells based on immunophenotype.

**What does this study add?**

- We present a novel semi‐automated workflow for the isolation of circulating fetal trophoblasts and single‐cell genomic profiling of submicroscopic variants.
- With this methodology, the feasibility of fetal genetic confirmation of each individual cell, a limit of detection (LoD) for CNVs down to 800Kb and the assessment of zygosity, fetal sex and genomic profiling in the same cell in twin pregnancies was demonstrated.

## 1 INTRODUCTION

With the feasibility and widespread clinical introduction of cell‐free DNA (cfDNA) testing for prenatal aneuploidy screening over the past decade,^1^ interest in the noninvasive screening of pathogenic genomic copy number variants (pCNVs) other than common trisomies has emerged. To a large degree, this has been stimulated by the burgeoning realization that pCNVs account for a significant burden of human morbidity and mortality and an overall cumulative higher prevalence than is accounted for by aneuploidy alone.^2^, ^3^, ^4^ Indeed, the increasing utilization of chromosomal microarray (CMA) as a higher resolution alternative to conventional karyotype for invasive prenatal genomic analysis, with or without the finding of fetal structural anomalies, has demonstrated the importance of CNVs in this regard.^2^, ^3^, ^4^, ^5^

Despite interest and clinical offerings (both commercial and public) of screening for CNVs with cfDNA‐based methodologies, a true noninvasive comprehensive profiling of the fetal genome for microdeletions and microduplications is not possible due to limitations related to the poor resolution of cfDNA‐based testing for imbalances of smaller size.^6^, ^7^, ^8^, ^9^, ^10^ Therefore, a low‐risk result with cfDNA test is still associated with a consistent residual risk. Most of these limitations, however, could be resolved by the noninvasive capture of intact fetal cells and a subsequent analysis of the pure whole fetal genome. Indeed, the isolation of intact fetal cells from maternal circulation in a scalable and automated workflow would create feasibility around the potential for expanding screening to include pCNVs in a more robust manner than is currently possible with a maternal admixture of fragmented cfDNA retrieved from maternal plasma.

Of course, interest in the noninvasive capture and interrogation of intact fetal cells have been a significant work in progress and promise for at least the past 20 years for noninvasive prenatal testing,^11^ but have been technically challenging due to a number of issues related to their scarcity, absence of robust and scalable capture methodologies and individual sample variability.^12^ Circulating extravillous trophoblasts (cEVTs) were the first fetal cell type found in maternal blood when clusters of trophoblasts in the pulmonary capillaries of women who had died of eclampsia were identified.^13^ Due to their presence in maternal circulation during the first trimester of pregnancy, they became an attractive target for noninvasive prenatal testing (NIPT).^14^, ^15^

Previous investigations of methods for isolation of cEVTs resulted in laborious protocols with inconsistent and insufficient cell recovery^16^ as the identification of cEVTs by manual needle‐based cell picking or laser capture microdissection under the microscope is highly operator‐dependent.^15^, ^17^, ^18^

Recently, advances have been made in the single‐cell sorting of cEVTs using flow cytometry, followed by Short‐Tandem Repeats (STR) analysis.^19^ However, fetal confirmation by STR analysis requires a separate additional test, which increases costs, hands‐on‐time and operator manipulation. In the context of a routine clinical testing application, manual protocols are not suitable or provide adequate reproducibility. Therefore, automated and high‐throughput cell‐recovery procedures are still needed.

Here, we present a multi‐step workflow for the semi‐automatic isolation and analysis of cEVTs and the use of a proprietary integrated algorithm for fetal cell origin genetic confirmation. We describe our early experience in a proof‐of‐concept study with sample variability, number of isolated cells and CNV resolution with our current downstream single‐cell analysis.

## 2 MATERIALS AND METHODS

### 2.1 Limit of detection and analytical performance for copy number variant

Copy number variant LoD was analytically assessed with two sets of experiments using the Coriell panel of human‐derived cell lines (Catalog ID: CNVPANEL01) harboring well‐characterized segmental imbalances of different sizes.

The first set of experiments was carried out on genomic DNA (gDNA) from 43 cell lines analyzed in triplicate for a total 127 tests on 58 target imbalances ranging from 100Kb to 155Mb in size (Supplementary Table 1). For each cell line, three aliquots of 1 ng human gDNA were whole‐genome amplified (WGA) using the *Ampli*1 WGA kit and the libraries obtained with *Ampli*1 LowPass kit (Menarini Silicon Biosystems) were sequenced on an Illumina platform. All CNVs were then identified with an in‐house‐developed copy‐number pipeline (details in Supplementary Methods), starting from FASTQ files downsampled to 3M reads.

In the second set of experiments, single cells from 5 Coriell cell lines harboring micro‐imbalances ranging from 200Kb to 3.6Mb (size range spanning the resolution determined with previous gDNA experiments) were analyzed after isolation by flow cytometry. To evaluate the reproducibility for imbalance detection, a range of 10–17 single cells were tested for each cell line (total single cells tested = 73). Sequence profile quality parameters together with visual inspection were used to exclude low‐quality profiles or apoptotic (chaotic profiles indicative of genomic degradation) and S‐phase cells (partially replicated genomes with short and evenly distributed losses). Methods for the calculation of analytical performance are described in Supplementary methods.

### 2.2 Patient recruitment and blood samples

Women aged ≥18 years, with singleton or twin pregnancies, presenting for routine prenatal care at two public‐health maternity hospitals (Fondazione IRCCS Ca’ Granda Ospedale Maggiore Policlinico in Milan and MBBM Foundation Onlus at San Gerardo Hospital, Italy), were enrolled from October 2019 until December 2020. The study protocol was approved by the local Institutional Review Boards at both study sites (#1227 and #2648).

When an invasive fetal diagnostic procedure was clinically indicated, venipuncture was performed prior to the procedure. Twenty milliliters of maternal blood was collected in two 10 ml CellSave preservative tubes (Menarini Silicon Biosystems), kept at room temperature until processing within 96h from sampling. Once cEVTs were sorted and analyzed, operator blinded results were ultimately compared to the genetic ‘truth’ defined by the clinical laboratory diagnosis (e.g. cytogenetic and/or microarray analysis).

### 2.3 Trophoblast enrichment and single‐cell sorting

**FIGURE 1:**
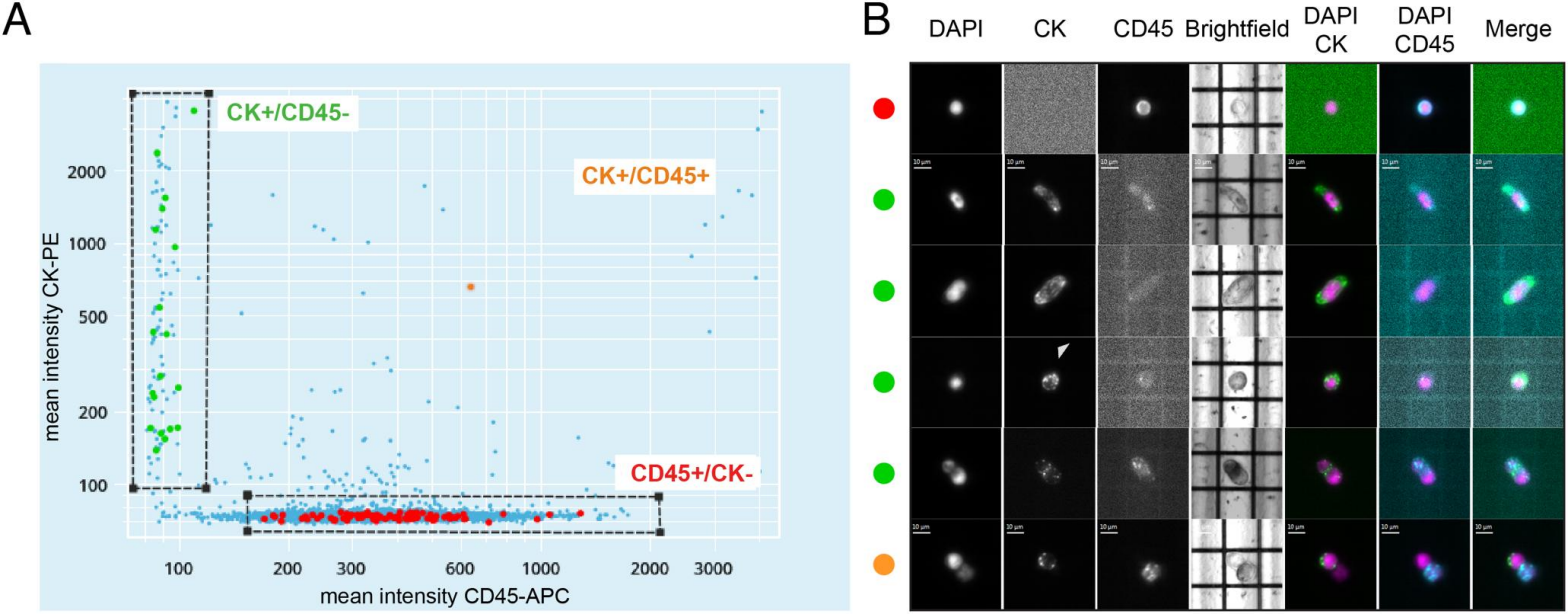
Single‐trophoblast selection and immunophenotypic staining. (A) Scatter plots of pan‐cytokeratin–phycoerythrin (CK‐PE) mean intensity (*y* axis) versus CD45–allophycocyanin (APC) mean intensity (*x* axis) of putative circulating extravillous trophoblasts (cEVTs) (CK+/CD45− cells), white blood cells (WBCs) (CK−/CD45+ cells) and a mixed cell (CK+/CD45+). Selected WBCs, cEVTs and mixed cells are highlighted in red, green and orange, respectively. (B) Image gallery of one representative WBC (side red dot), four representative cEVTs (side green dots) and a representative mixed cell (side orange dot); DAPI (4',6‐diamidino‐2‐phenylindole), CK, CD45 and brightfield single channels are visualized in grayscale; DAPI/CK, DAPI/CD45 and Merge combined channels are visualized as follows: DAPI signal in purple, CK signal in green and CD45 signal in cyan. The four representative cEVTs exhibit different typical morphological features from top to bottom: elongated cells with cytoplasmic processes, cells with an even distribution of cytoplasmic cytokeratin, so‐called “bubble” cells with cytokeratin high‐density spots (arrowhead) and CK+/CK+ doublets. Scale bars: 10 μm.

**FIGURE 2:**
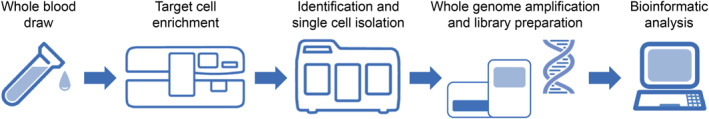
Experimental multi‐step workflow for single‐trophoblast selection, identification and sorting. Whole blood was enriched and stained using the CellTracks AutoPrep System. After sample preparation, DEPArray system was used for isolation of single circulating extravillous trophoblasts (cEVTs) and maternal white blood cells (WBCs) for downstream analysis.

Maternal blood samples were enriched for target fetal trophoblast cells using proprietary ferrofluid‐conjugated‐specific antibodies and a custom protocol on the automated CellTracks AutoPrep System (Menarini Silicon Biosystems). Automated cell staining and enrichment of the fetal trophoblasts (cEVTs) resulted in a sample that was loaded into the image‐based DEPArray system (Menarini Silicon Biosystems) for the analysis. The selection of cEVTs was done through the CellBrowser image‐analysis software (Menarini Silicon Biosystems) based on pan‐cytokeratin (CK) positivity and CD45 negativity as well as by morphological features (Figure 1A,B). Putative fetal trophoblasts were then automatically recovered as single cells for the downstream analysis. Maternal white blood cells were also recovered as controls. Each step and its respective processing time are shown in Table 1. The total time from initial blood processing to single‐cell recovery was less than 6 h. A schematic representation of the workflow is depicted in Figure 2.

**TABLE 1:** Steps and timing of the automated workflow for single‐cell recovery.

Automated step	Instrument	Processing time per sample
cEVT enrichment	CellTracks Autoprep	3 h
cEVT staining		
Cell load, scan and selection	DEParray	1.5 h
cEVT confirmation	DEParray—User interaction	10–20 min
Single cEVT recovery	DEPArray	1 h

### 2.4 Copy‐number profiles for aneuploidies and copy number variant identification on isolated circulating extravillous trophoblasts

After cell lysis of maternal white‐blood‐cell pools and single cEVTs, gDNA was amplified using the *Ampli*1 WGA kit and sequenced with the *Ampli*1 LowPass (Menarini Silicon Biosystems) for Illumina library preparation with a fully automated workflow on a STARLET Hamilton Liquid‐Handling Robot (Hamilton Bonaduz AG, Bonaduz, Switzerland). Then, library concentrations were normalized and sequenced on an Illumina NovaSeq 6000 platform (Illumina, San Diego, CA, USA), typically obtaining ∼5M reads per sample on average. Copy‐number profiles were obtained using an in‐house‐developed bioinformatic pipeline (details in Supplementary Methods).

### 2.5 Fetal‐origin confirmation and zygosity assessment in twin pregnancies

Fetal origin was confirmed by a single‐nulceotide polymorphism (SNP) based proprietary algorithm using sequencing data from maternal and fetal cells obtained for copy‐number profiling, also enabling zygosity assessment in twin pregnancies.

In samples from women not undergoing the invasive procedure, fetal origin was assessed through the allele comparison of STRs with the corresponding maternal DNA.

Short‐Tandem Repeat analysis on WGA products was also applied to confirm and validate fetal identity and twin zygosity as determined by the proprietary SNP‐based algorithm.

## 3 RESULTS

### 3.1 Limit of detection and analytical performance for copy number variant

**FIGURE 3:**
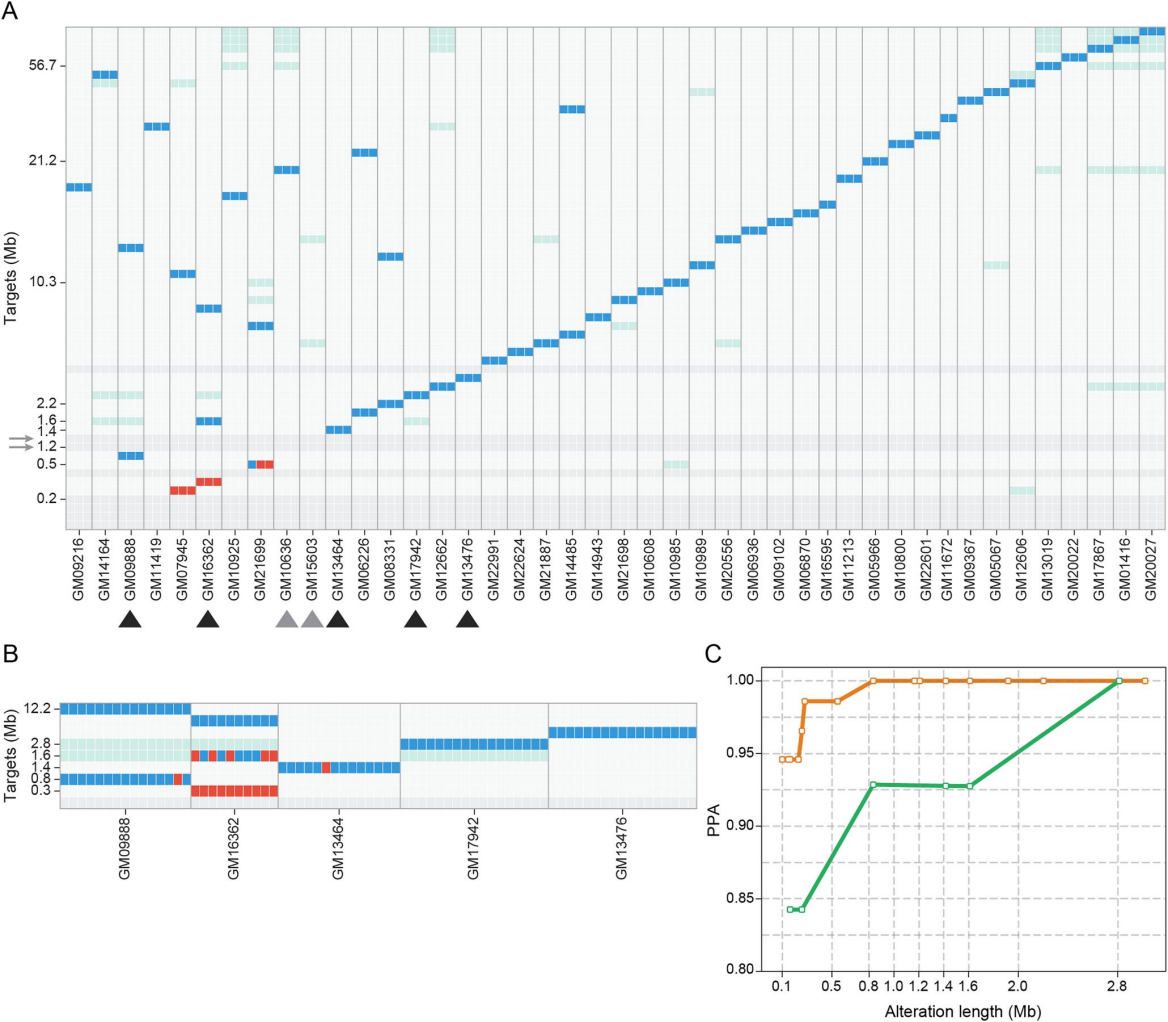
Copy number variant (CNV) limit of detection (LoD) experiments. (A) The Coriell targets have been arranged on the *y* axis in the ascending order according to their size, while the cell line samples (genomic DNA (gDNA))—3 replicates per cell line—have been ordered on the *x* axis according to the size of the targets expected in the cell line itself. Each squared cell represents the detection status of the single target in a single replicate with analytical true positives in blue (the copy‐number value for the x cell line in the y target matches the expected altered copy‐number), analytical false negatives in red (the copy‐number value for the x cell line in the y target does not match the expected altered copy‐number) and true negatives in light grey (the copy‐number value for the x cell line in the y target matches the expected normal value). Dark grey and light‐green boxes show, respectively, “no call” targets, characterized by a poor copy‐number signal, and “no eval” targets, in cell lines that harbor overlapping targets. (B) Single cells isolated from a subset of Coriell cell lines, chosen based on target size close to 1Mb, were assessed to confirm the resolution determined with gDNA using samples that simulate real cases (cell lines used are indicated by black arrowheads in panel A). (C) Trend of analytical sensitivity (Positive Percent Agreement (PPA)) according to increasing target length measured on the data described in panels A (orange line) and B (green line).

The two sets of experiments with Coriell cell lines both demonstrated an LoD down to 800Kb (Figure 3A,B) with a confirmation obtained by 3/3 of gDNA replicates and 14/15 tested cells at this size level. Moreover, no false negatives were detected for targets ≥800Kb with gDNA and >1.6Mb with single‐cell experiments (Figure 3C). These findings resulted in a 100% Positive Percent Agreement for CNVs down to this imbalance size.

No false positives were observed in the tested targets, indicating a striking calling reliability at imbalance‐prone critical regions. However, a low number of false positives (∼0.26 per gDNA library and ∼0.56 per single cell) have been observed randomly distributed along the whole genome and the vast majority of them are lower than 1Mb in size (∼82% and ∼73% of false positives in gDNA and single‐cell samples, respectively) (Supplementary Figure 1).

A specific genomic target of 1.6Mb (cell line GM16362) was detected in 50% replicates in single‐cell experiments, while it was correctly detected in all gDNA replicates (Figure 2A,B). This target is located in the pericentromeric region of 22q suggesting a possible positional bias that, in combination with the small size of the imbalance, makes its identification challenging (Supplementary Figure 2A). Additional examples of expected alterations identified with our approach are shown in Supplementary Figure 2B and 2C, where the 800 Kb deletion on chr14 for GM09888 and of 2.8 Mb deletion on chr22 for GM17942 (DiGeorge Syndrome) are shown.

Of note, two expected deletions of 1.2 and 1.3Mb (GM15603 and GM10636) were not identified by experiments with gDNA as they are located in low‐mappability or pericentromeric regions of chromosome 2 (2q13) and 15 (15q11.2) characterized by repetitive sequences and thus considered not callable (grey boxes highlighted with grey arrows in Figure 2A).

### 3.2 Circulating extravillous trophoblast isolation from maternal blood samples and fetal identity genetic confirmation

**FIGURE 4:**
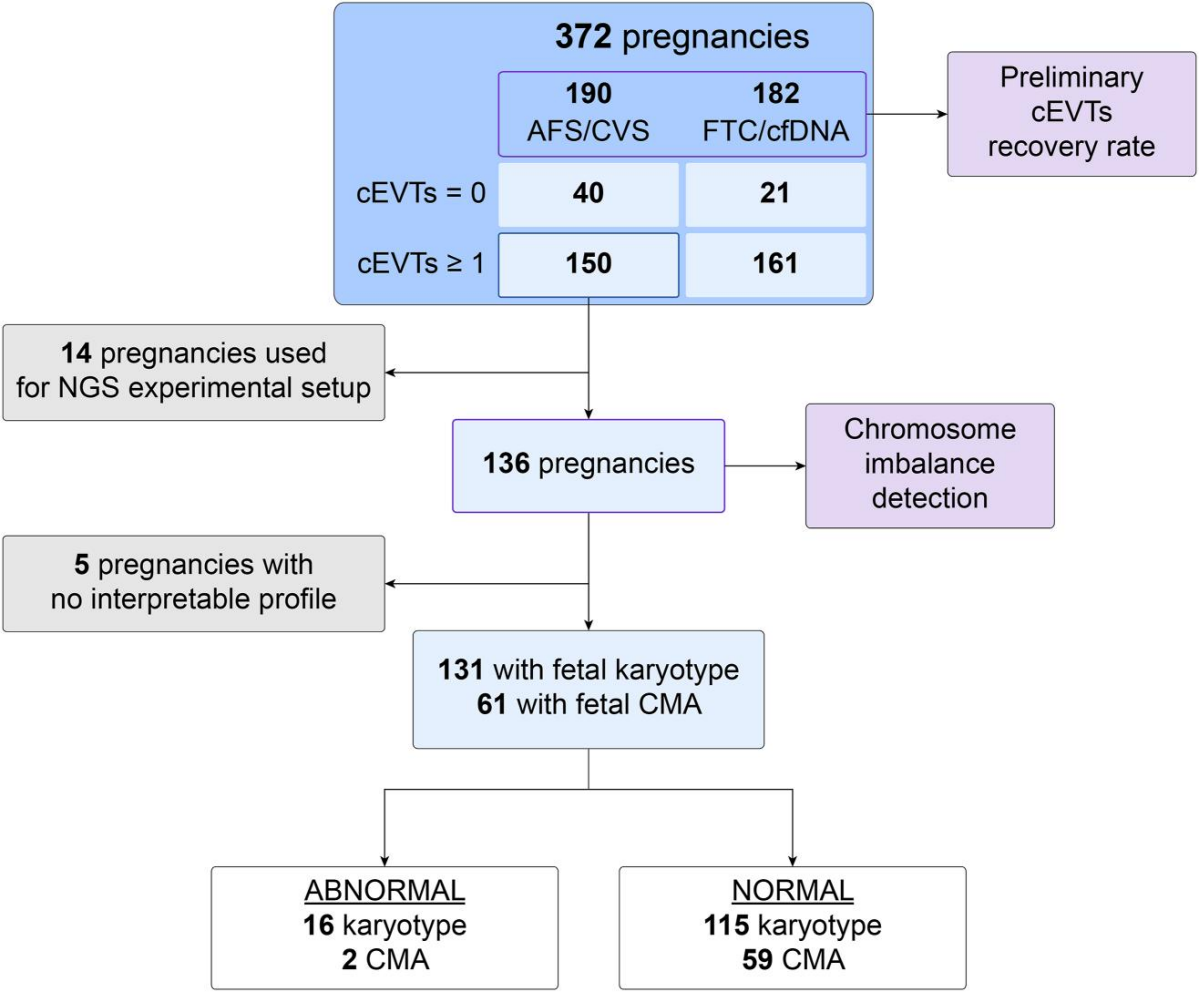
Study structure and enrollment. AFS, Amniocentesis; cEVTs, circulating Extravillous Trophoblasts; cfDNA, high risk cell‐free DNA test; CMA, chromosomal microarray analysis; CVS, Chorionic Villus Sampling; FCT, High‐risk First trimester Combined Test; NGS, Next‐Generation Sequencing.

**FIGURE 5:**
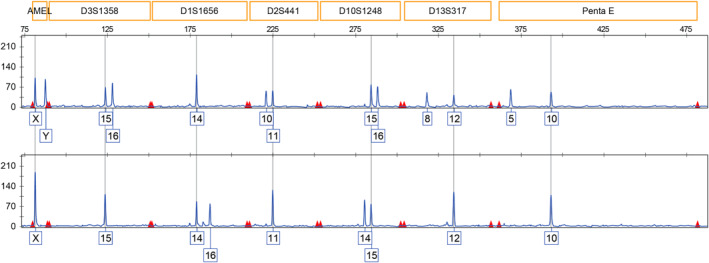
Maternal‐to‐fetal allele comparison of Short‐Tandem Repeats (STRs). Examples of a fetal (top) and maternal (bottom) STR profiles are shown.

**FIGURE 6:**
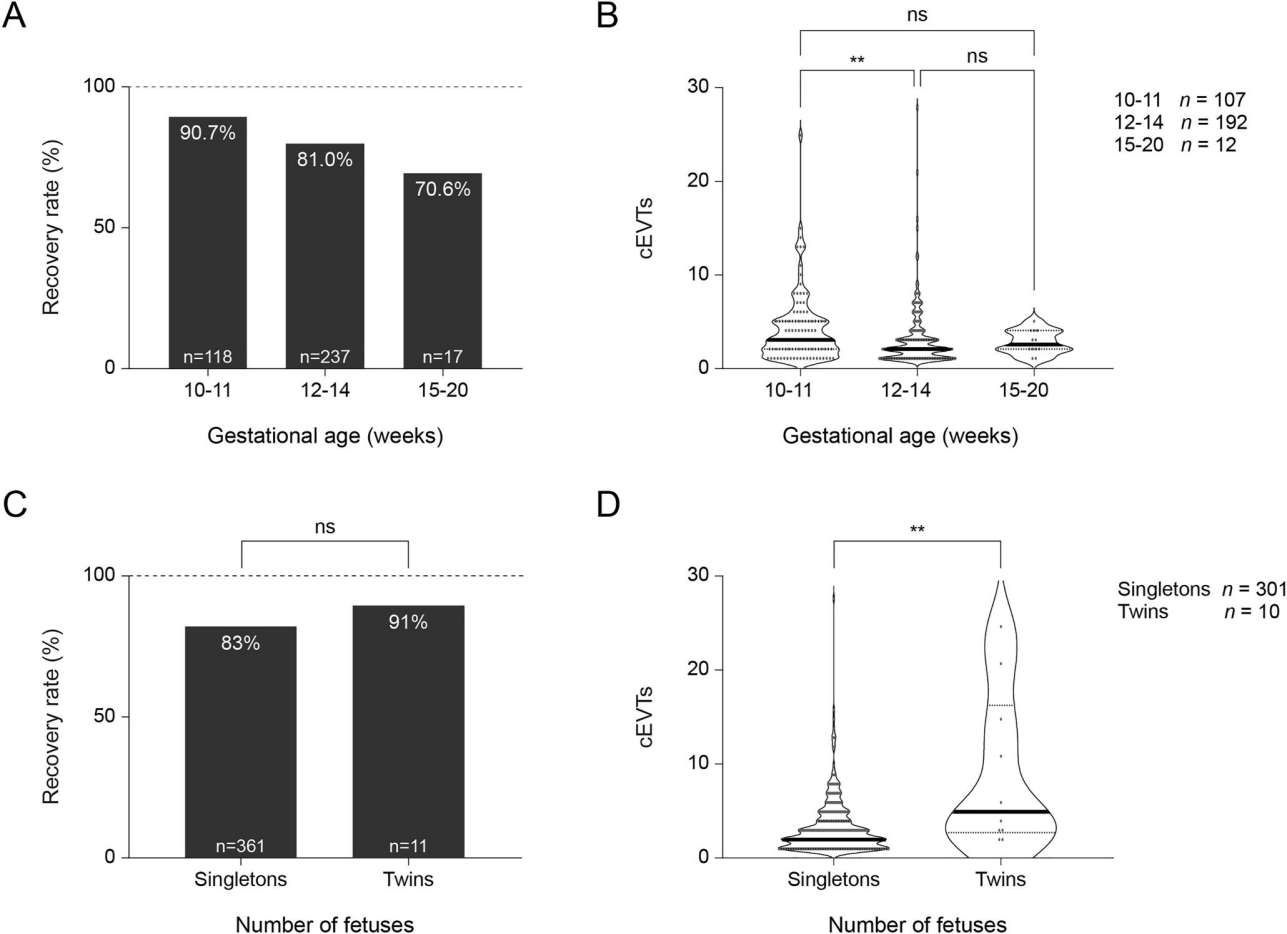
Recovery rate and number of trophoblasts identified. (A and B) Patients were analyzed according to gestational age range: 10–11 weeks, 12–14 weeks and 15–20 weeks. (A) Recovery rate and (B) violin plots of circulating extravillous trophoblasts (cEVTs) (solid lines represent median values) in patients with at least one cEVT isolated. A significant linear trend was observed between gestational weeks and recovery rate (chi‐square test for trend, *p* < 0.01). The number of recovered cEVTs was significantly different comparing 10–11 weeks and 12–14 weeks (Kruskal–Wallis test followed by Dunn's multiple‐comparison test, **p < 0.01). (C and D) Patients were analyzed according to the number of fetuses: Singletons versus Twins. Recovery rate (C) and violin plots (D) of cEVTs (solid lines represent median values) in patients with at least one cEVT isolated. For 365 out of 372 patients, the number of fetuses and fetal gender was available. Patients with an unknown number of fetuses and/or fetal gender were not included. The number of cEVTs is significantly higher in twins compared to singletons (nonparametric Mann–Whitney *U* test, *p* < 0.01).

To validate the method on a clinical cohort, 372 patients were enrolled during the study period. The study design is provided in Figure 4 and a summary of the demographic information and indications is reported in Table 2. In 311 of the 372 maternal blood samples (83.6%), at least one cEVT was recovered for a total of 1084 individual cEVTs whose fetal identity was genetically confirmed by both SNP‐based method and STR assays (Figure 5). The recovery of at least one cEVT was 90.7% at 10–11 weeks with 4.2 cells per sample (*n* = 118), 81.0% at 12–14 weeks with 3.1 cells per sample (*n* = 237) and 70.6% at 15–20 weeks with 2.8 cells per sample (*n* = 17) (Figure 6A), showing a significant linear trend between gestational‐age and cEVT recovery (Cochran–Armitage test for trend, *p* < 0.01). In samples with cEVT recovery, the mean number of recovered cells was 3.5 per sample (mean ± SD = 3.5 ± 3.4, Figure 6B), variable depending on the gestational age at sampling. The number of recovered cEVTs was significantly different compared with 10–11 weeks and 12–14 weeks (*p* < 0.01); no significant difference was observed in comparison with the 15‐ to 20‐week group.

**TABLE 2:** Demographic information of the study cohort

Number of pregnant women	372
Mean whole blood volume (min–max)	17.57 ml (9.5–20.5 ml)
Mean gestational age (min–max)	12.20 weeks (10–20 weeks)
Fetal gender	195 F, 181 M, 7 not known
Number of fetuses	361 singletons, 11 twins
Mean maternal age (min–max)	35.30 years (20–47 years)
Mean maternal BMI (min–max)	22.79 kg/m^2^ (14.7–40.0 kg/m2)
Referral reason	
FCT	36.83% (*n* = 137)
cfDNA test	12.10% (*n* = 45)
CVS	46.51% (*n* = 173)
AFS	4.57% (*n* = 17)
**Indications** for CVS and AFS	
Advanced maternal age	21.11% (*n* = 42)
Abnormal ultrasound findings	5.26% (*n* = 10)
Positive FCT	53.68% (*n* = 102)
Positive cfDNA	4.21% (*n* = 8)
Other^a^	14.74% (*n* = 28)
Gestational age (range)	
10–11 weeks	31.72% (*n* = 118)
12–14 weeks	63.71% (*n* = 237)
15–20 weeks	4.57% (*n* = 17)

Abbreviations: AFS, Amniocentesis; BMI, Body Mass Index; cfDNA, high‐risk cell‐free DNA; CVS, Chorionic Villus Sampling; FCT, high‐risk First trimester Combined Test.

^a^ Includes: Parent carrier of a chromosomal abnormality, prior affected pregnancy, and risk for monogenic disorders.

**FIGURE 7:**
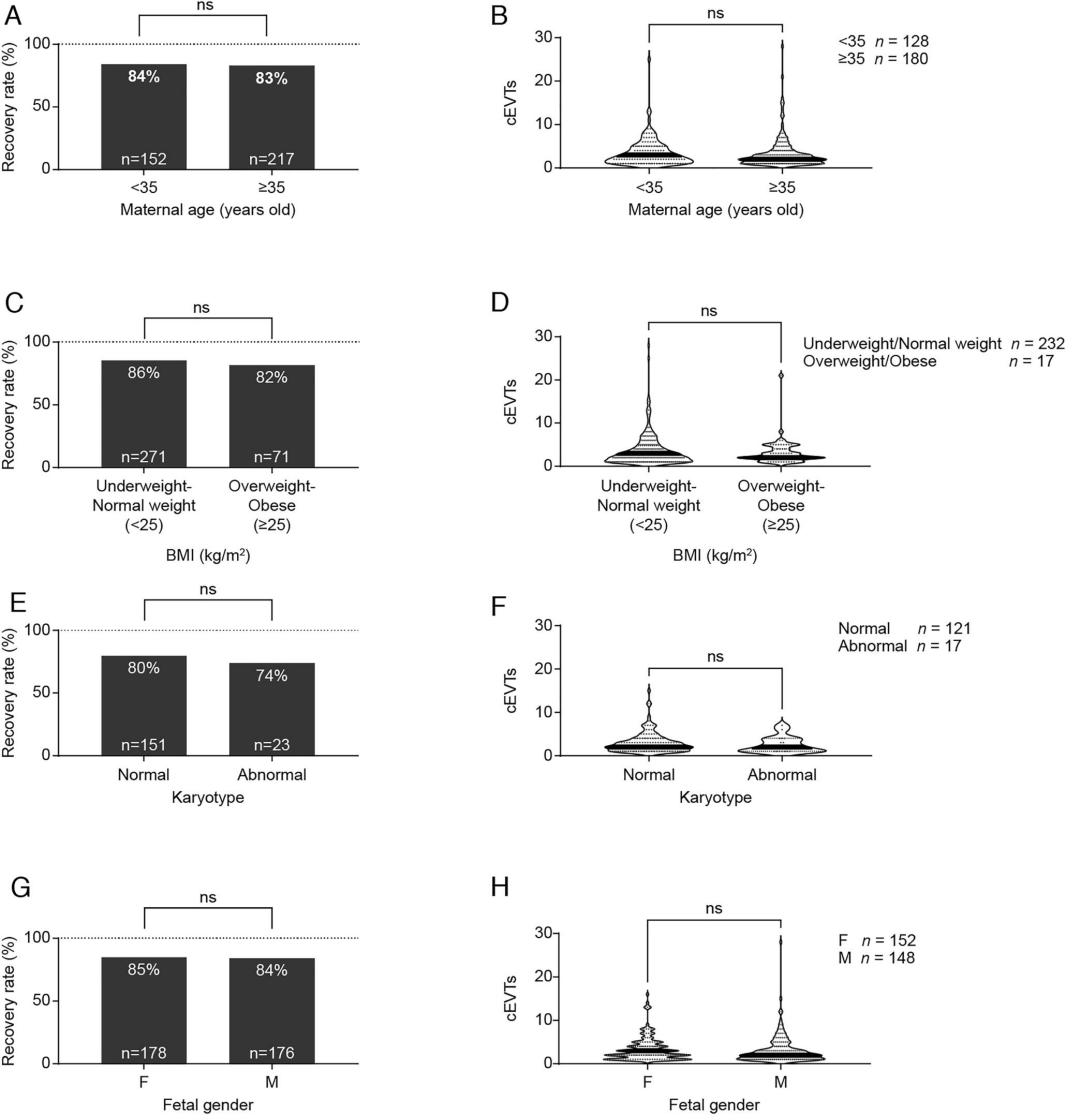
Recovery rate and circulating extravillous trophoblasts (cEVTs) of patients classified according to different characteristics. Recovery rate (A, C, E, and G) and violin plots of recovered cEVTs (B, D, F and H, solid lines represent median values). (A and B) Maternal age: <35 years versus ≥ 35 years; for 369 out of 372 patients, maternal age was available. Patients with unknown maternal age were not included. (C and D) BMI: <25 kg/m2 (underweight/normal weight) versus ≥ 25 kg/m2 (overweight/obese). For 342 out of 372 patients, body mass index (BMI) was available. Patients with unknown BMI were not included. (E and F) Karyotype: Normal versus Abnormal (monosomy X (MX) *n* = 1; trisomy 18 (T18) *n* = 3; T21+mosaic T18 *n* = 1; trisomy 21 (T21) *n* = 18). For 185 out of 372 patients, fetal karyotype was available. Patients with unknown karyotype and twins were not included. (G and H) Fetal gender: F (female) versus M (male). For 354 out of 372 patients, fetal gender was available. Patients with unknown fetal gender and twins were not included.

Maternal age, body mass index (BMI), fetal gender and chromosomal abnormalities were not significantly associated with cell recovery rate or the number of isolated cEVTs (Figure 7).

### 3.3 Single‐cell Next‐Generation Sequencing analysis of isolated circulating extravillous trophoblasts

One hundred fifty of the 311 patients with cEVT recovery underwent an invasive procedure (Figure 4). Fourteen samples were used for Next‐Generation Sequencing (NGS) experimental protocol development, and thus, excluded from the copy‐number analysis. Therefore, 136 of the 150 samples (total 432 cEVTs) underwent copy‐number profiling. Ultimately, the comparison with fetal clinical diagnostic karyotyping results was possible for 131 women, as interpretable profiles could not be obtained for five samples where only one cEVT was recovered due to either low‐quality sequencing libraries, apoptotic‐like cells or S‐phase mixed recoveries. In 61 of the 131 samples, a supplementary fetal CMA was available for submicroscopic imbalance comparison. Overall, a total of 29/432 (6.7%) single‐cell profiles were uninterpretable. Supplementary Table 2 reports detailed description of the cEVTs profiles.

### 3.4 Aneuploidy classification

**FIGURE 8:**
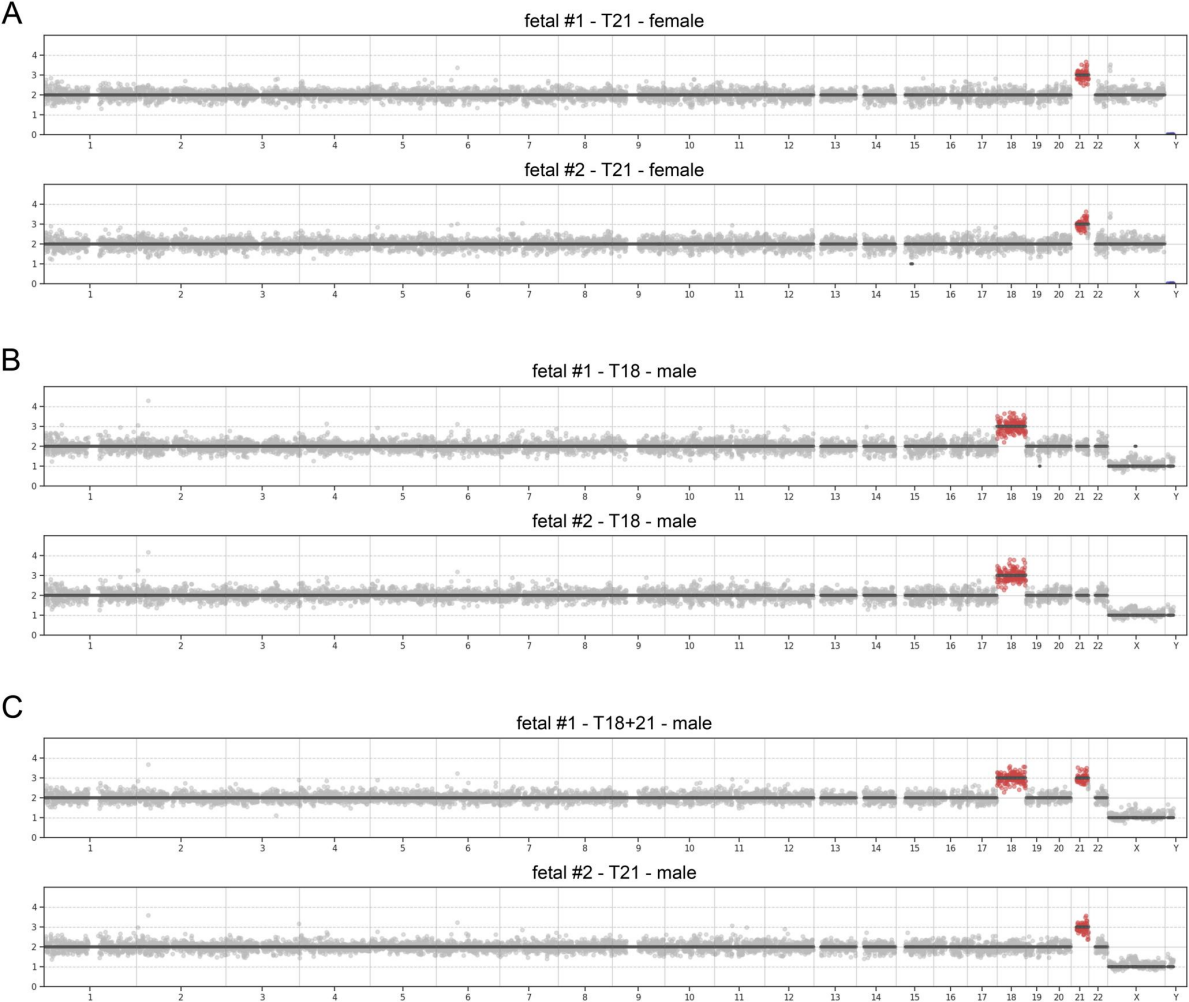
Copy‐number profiling of fetal cells. Absolute copy‐number profiles in the fetal cells isolated from three pregnant women. This figure shows one case of trisomy 21 (T21), one with trisomy 18 (T18) and one case with both T21 with a mosaic T18. In this case, the CVS‐derived karotype analysis identified a 47,XY,+21 karyotype in cytotrophoblasts (direct prep) and a 48,XY,+18,+21 karyotype in mesenchymal cells (long term culture). These data suggest the presence of a 48,XY,+18,+21 cell line in the cytotrophoblasts that were undetected by the Chorionic Villus Sampling (CVS) direct‐preparation cytogenetic analysis. (A) Copy‐number profiles of two different fetal cells of case M019, showing a T21 in a female fetus. (B) Copy‐number profiles of two cells with T18 in a male fetus (M057). (C) Two different fetal cells from the same pregnant woman (Case M115) showing the presence of T21 with a mosaic T18. T21, trisomy 21; T18, trisomy 18.

In the group with the fetal invasive cytogenetic analysis, there were 16 abnormal and 115 normal karyotype results. Single‐cell analysis (Figure 8) of cEVTs provided full concordance with normal and abnormal cytogenetic results except for one case with a normal fetal karyotype result in which a T16 was identified in all cEVTs analyzed (3/3), therefore identifying a confined placental mosaicism (CPM) for T16. Interestingly, in case #M115, Chorionic Villus Sampling (CVS) cytogenetic analysis detected trisomy 21 (T21) in cytotrophoblast and a double T21 and 18 in mesenchyme. Single‐cell analysis confirmed the T21, and in addition, detected the double trisomy in circulating trophoblasts (Figure 8C and Supplementary Table 3). Of note, a single case of 69,XXY karyotype was also identified.

### 3.5 Copy number variant detection

In the cohort of 61 cases with fetal CMA analysis, there were 2 cases with pathogenic CNVs and 59 with normal CMA results. Single‐cell cEVTs analysis demonstrated complete CNV concordance with clinical fetal CMA results. In the group with normal CMA results, there were 6 (likely) benign CNVs, all copy number gains, ranging from 900 Kb to 3 Mb in size (Table 3). They were all identified by single‐cell analysis in cEVTs.

**TABLE 3:** Characteristics of the 6 benign duplications detected with circulating extravillous trophoblasts (cEVTs) single‐cell analysis ranging from 3Mb to 900Kb

Patient ID	Fetus sex	Genomic location	Copy number	Length (Mb)	Cells with alteration^a^
M031	XX	chrX:6400000–8100000	3	1.7	1 (100%)^b^
M055	XY	chr4:68000000–71000000	3	3	1 (100%)
M074	XY	chr17:63600000–64600000	3	1	2 (66.7%)
M113	XX	chr7:125200000–126100000	3	0.9	2 (100%)
M143	XX	chr18:47600000–49000000	3	1.4	2 (100%)
M156	XX	chr13:23500000–25400000	3	1.9	3 (100%)

^a^ Percentages calculated relative to the total number of high‐quality fetal profiles available.

^b^ Twin pregnancy with 2 fetal cells were recovered, 1 from each fetus. Alteration was revealed in both cells; however, the cell from twin B was in S phase. Therefore, the call out of the microduplication was not reliable.

**FIGURE 9:**
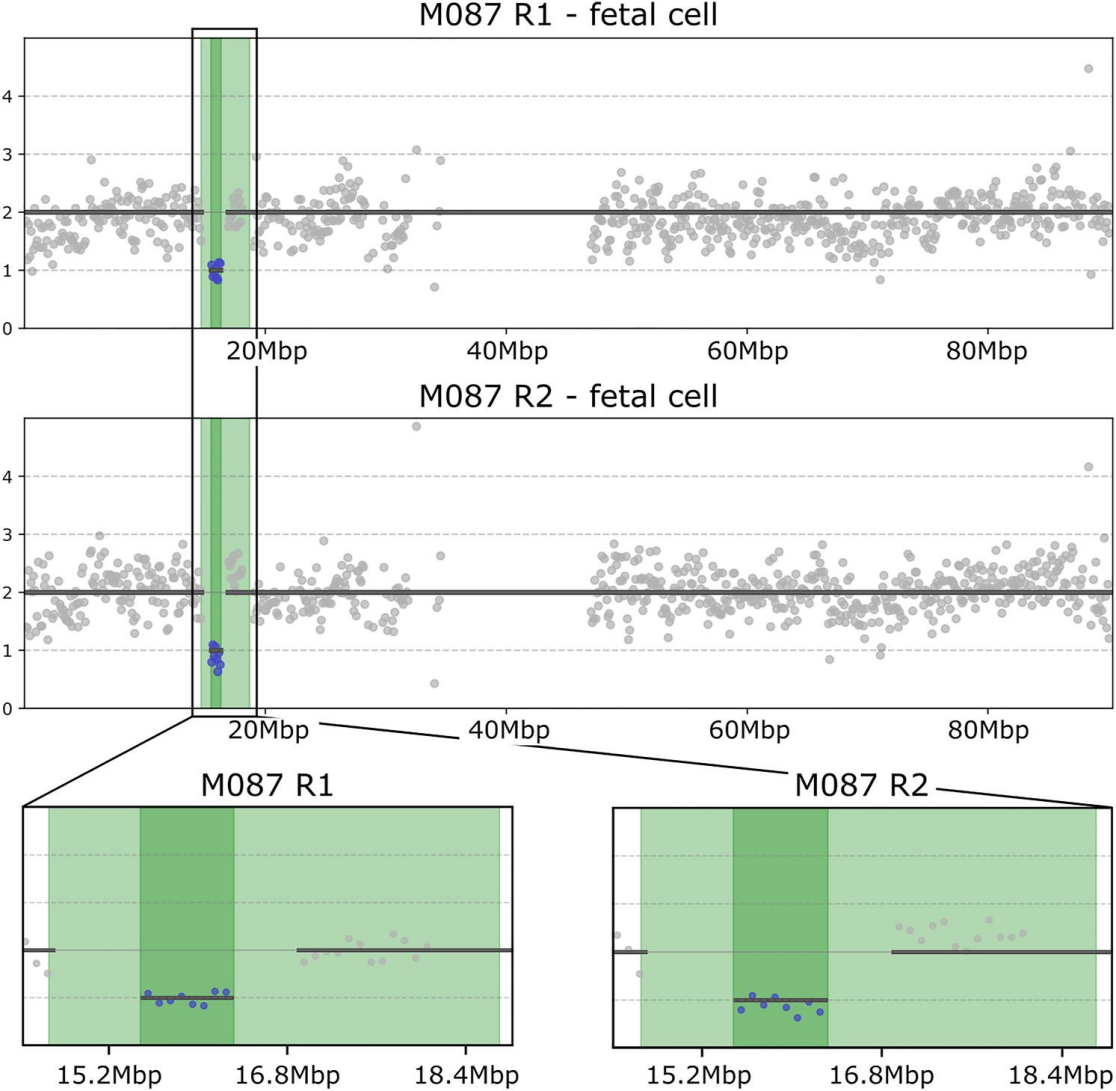
16p13.11 microdeletion syndrome detected by chromosomal microarray (CMA) and Next‐Generation Sequencing (NGS) on single‐cell circulating extravillous trophoblasts (cEVTs) analysis. Profile of the 800Kb deletion within the 16p13.11 region (chr16:15500000–16300000) in 2 out of 2 cEVTs recovered from the same pregnancy (case M087). Highlighted in light green is the largest 16p13.11 region involved in the syndrome (3.3 Mb); while in dark green is depicted its critical region.

One fetal pCNVs detected on CMA was a 4q26 duplication of 1.5Mb that could not be confirmed by single‐cell analysis as the two isolated cEVTs were low quality in one case and an S‐phase cell in the other. Thus, they were not suitable for CNV detection but only for aneuploidy classification. The second fetal pCNV found on CMA was a 16p13.11 deletion of 800kb that was detected by single‐cell analysis in 2/2 isolated cEVTs (Figure 9).

### 3.6 Twin pregnancies

In this series, a total of 11 twin pregnancies were enrolled (Table 4) and cEVT isolation was possible in 10/11 cases. A significant increase (*p* < 0.01) in the number of recovered cells was observed in comparison to singletons (Figure 6C,D). The three Monochorionic (MC) pregnancies showed only one fetal genetic profile; 2/4 known Dizygotic (DZ) pregnancies and 2/2 Dichorionic (DC) pregnancies showed two fetal genetic profiles. In the remaining two DZ pregnancies with one fetal genetic profile, 2 and 3 cEVTs were isolated, respectively. In one case, confirmation of zygosity was not available but two fetal genetic profiles were detected. Copy‐number profiling of cEVTs was performed in 6/7 twin pregnancies that had undergone invasive prenatal diagnosis. Trisomy 21 was identified on cEVT analysis in a MC twin pregnancy; the five remaining cases showed a normal profile. These results were concordant with that of fetal karyotyping and CMA analysis.

**TABLE 4:** Zygosity results in twin pregnancies

Patient ID	N° fetal cells analyzed	Gender of twins (Twin A | Twin B)	Zygosity or Chorionicity^a^	N° fetal genetic profiles identified
M012	3	F|F	DZ	1
M019	6	F|F	MC	1
M031	2	F|F	DC	2
M034	21	F|F	MC	1
M040	3	M|F	DZ	2
M082	2	M|F	DZ	1
M144	0	M|F	DZ	nd
M184	15	M|F	DZ	2
M226	25	F|F	MC	1
M326	11	na	DC	2
M339	4	F|F	na	2

Abbreviations: DC, Dichorionic; DZ, Dizygotic; F, Female; M, Male; MC, Monochorionic; MZ, Monozygotic; na, not available; nd, not defined.

^a^ Defined by US scan and/or cytogenetic result and/or fetal gender and/or zygosity testing on CVS/AFS.

## 4 DISCUSSION

The semi‐automated methodology presented here for the isolation and single‐cell analysis of cEVTS from maternal blood supports the feasibility and scalability of a cell‐based noninvasive prenatal test for fetal genomic profiling down to ∼1Mb in size. The goal of our work was to develop a new method that, different from others reported,^16^, ^17^, ^18^, ^19^ would (i) reduce hands‐on manipulation of isolated fetal cells and (ii) both demonstrate the fetal origin by genetic confirmation (not only by immunophenotype) and perform fetal genome profiling for aneuploidy and subchromosomal variant in each isolated single cell. Current work is in progress to both validate our current workflow on a large patient cohort (over 1500 patients) to a single‐cell analytical LoD of ∼1Mb and to increase the overall number of samples with recovery and the number of cEVTs isolated per sample. In addition, as the workflow used for this study still includes manual steps (e.g. blood sample preparation, moving of the sample(s) between different automation steps) and with DEPArray one chip/sample, efforts are currently ongoing to increase both the level of automation and throughput.

Of the 372 patients recruited for our method validation study, about half underwent an invasive fetal diagnostic procedure. Ultimately, there were 131 cases with karyotype analysis that could be directly compared to analyzed isolated trophoblasts, and 61 of them had a clinical fetal CMA result. Our cEVT single‐cell analysis showed concordance with fetal karyotype results except for one case with normal cytogenetic analysis on amniocytes in which the cEVTs analysis showed a CPM for T16. In samples where isolated single cells could be analyzed for CNVs, there was also full concordance with fetal CMA results. For one case, where the quality of the cEVTs isolated (2/2) was only suitable for aneuploidy classification, there was a pCNV (duplication) of chromosome four of 1.5Mb on the fetal invasive sample analysis. Benign or likely benign duplications from 900Kb to 3Mb in size identified by CMA on CVS were all identified by our single‐cell method. Of note, the case with pathogenic 16p13.11 deletion of 800kb allowed the confirmation on cEVTs of the LoD of 800Kb. Given the characteristics of the patient group, sample size and utilization of fetal CMA in these particular institutions, there was little opportunity to evaluate the presence of pathogenic CNVs in our patient samples. Our current efforts are focused on accessing patient samples with pathogenic CNVs.

The release of trophoblasts into the maternal circulation is thought to be a result of their active proliferation during the initiation of invasion of the uterine arteries, followed by a progressive increase in apoptosis after the conclusion of trophoblast invasion.^20^, ^21^, ^22^, ^23^ We have observed that circulating trophoblast recovery is unlikely to be influenced by maternal BMI, a known factor in test failure with cfDNA prenatal testing modalities as increasing BMI is associated with a reduction in the fetal fraction of DNA.^24^ However, the number of cEVTs recovered per sample in our study cohort decreased with advancing gestational age (higher in the 10–11‐week group vs. 12–14‐week group). This finding is consistent with a previous observation resulting from microscope‐identified cells of relatively high numbers of cEVTs at early gestational age.^25^ The wide range of values for numbers of isolated cEVTs per patient (0–28) indicates that there is likely to be considerable variability between individuals. We did not observe any significant association between either the number of cEVTs or the recovery rate and factors, such as maternal age or BMI, fetal sex or karyotype; but we did observe a significant difference in the number of isolated cEVTs between women with twin and singleton pregnancies as described by others.^25^

A biological phenomenon that has been seen in the analysis of trophoblast cells through a direct CVS cytogenetic analysis is feto‐placental mosaicism,^26^ mainly involving whole‐chromosome aneuploidies. Therefore, potential discordant results for aneuploidies between cEVTs and fetal karyotype on amniocytes are possible, even when a fully abnormal or normal karyotype is detected on trophoblasts via analysis of cEVTs. In fact, based on the cytogenetic experience with CVS, these discordancies would be expected to be more likely with monosomy X and rare autosomal trisomies (RATs) rather than with T21, 18, 13.^27^ Indeed, we found a case where a full T16 was identified in all cEVTs and the karyotype from amniocentesis (AFS) was normal, suggesting the presence of a CPM for T16. Notably, when T16 is identified in the cytotrophoblast by CVS cytogenetic analysis, the likelihood of its fetal confirmation on AFS is only ∼16%.^28^ In addition, when a RAT is detected in all cytotrophoblasts by direct preparation alone (without analysis of long‐term cultures), the associated false‐discovery rate is 56.52%.^27^

A limitation for CNV detection in single‐cell analysis is the possibility to recover cells during apoptosis with low‐quality not analyzable DNA or in mitotic S phase. However, we have been able to call aneuploidy in S‐phase cells. These features underline the importance of recovering multiple cells from each sample in order to minimize inconclusive results or with assessment for aneuploidy only. A possible strategy to manage these situations could include increasing the volume of blood drawn or processed initially.

This study demonstrates our ability to overcome several important challenges of fetal cell‐based testing in addition to the innovation in automation. An important feature of our workflow relates to the molecular genetic confirmation of the fetal origin of isolated cells. We accomplished this with both STRs comparison and with the development of a proprietary algorithm based on SNP analysis. Using the same algorithm, the separate analysis of each individual circulating cell also enables the detection of distinct genetic signatures in the case of DZ twins. Single‐cell analysis of cEVTs has, therefore, the potential for discrimination between mono‐ and DZ twins for the identification of vanishing twins and for the analysis of higher‐order multiple fetuses. Although zygosity testing by cell‐based NIPT would not be a replacement for routine first‐trimester ultrasound evaluation of chorionicity, there are specific cases where additional information on zygosity may be helpful for pregnancy management, such as when there is fetal anatomic abnormality or growth restriction in only one of the two fetuses. After monozygosity is established, restricting CVS or amniocentesis to only the anatomically abnormal fetus might be the preferred approach.^29^

For subchromosomal profiling of the fetal genome at the single‐cell level, experiments on CNV containing cell lines and the results of this study demonstrate that our current LoD using low‐coverage NGS is 800Kb. This level of detection would allow the identification of most known pathogenic CNVs.^2^ Improvements of our workflow are ongoing to increase the resolution for CNVs in clinically relevant regions with low mappability/low complexity, confirm our LoD and evaluate the rate of false positives with regard to smaller imbalances on isolated cEVTs from patient samples.

### 4.1 Advantages over cell‐free DNA‐based NIPT

Isolated single‐cell‐analyzed trophoblasts as a fetal DNA source avoids the confounding issues inherent in maternal plasma cfDNA analysis as cell‐based methodology avoids maternal DNA contamination as we have shown. Therefore, this approach would allow access to noninvasive prenatal testing in women with a previous allogeneic organ transplant/transfusion and with known chromosome abnormalities (germinal or somatic) avoiding unwanted incidental/secondary findings related to maternal genetic make‐up. In addition, our study suggests that cell recovery rate or the number of isolated cEVTs are not influenced by maternal BMI and fetal chromosomal abnormalities. This allows a noninvasive fetal genetic assessment to women with high BMI, which is typically associated with an increased no‐result rate with cfDNA testing.^30^, ^31^, ^32^, ^33^ Finally, cEVTs from in vitro fertilization (IVF) pregnancies obtained with egg or embryo donor procedures can undergo CNV profiling. In these IVF pregnancies, SNP‐based cfDNA testing is limited only to common trisomies, likely because of SNP profile crowding. Counting‐based methods do not assess genotype features, and therefore, these samples are analyzed. However, clinical validation studies on this pregnancy population are still missing.

In the case of twins, we have shown that single‐cell analysis can report on each fetal genome separately, thereby allowing the evaluation of fetal origin confirmation, zygosity, fetal sex and the copy number profiling in the same cell. With cfDNA‐based testing based on counting methods, twins cannot be genetically recognized individually as only the total fetal fraction can be evaluated, and the aneuploidy risk score and fetal sex probabilty are provided for the whole pregnancy. With cfDNA testing based on SNPs, individual fetal fractions, zygosity and fetal sex can be provided individually. However, a single aneuploidy risk score is provided for the whole pregnancy and the SNP profile of DZ twin pregnancies cannot be discriminated from that of a triploid unless there is the support of an ultrasound evaluation.^29^ Overall, the performance of cfDNA screening in multiple pregnancies of higher order than two is quite limited and needs further research.^34^

Compared with cfDNA testing, the noninvasive analysis of pure unfragmented gDNA from the conceptus is highly advantageous, as it can achieve true high‐resolution noninvasive comprehensive fetal genome profiling for detection of pathogenic submicroscopic imbalances. CfDNA testing based on genome‐wide assessment by shallow sequencing has a low resolution for known recurrent pathogenic submicroscopic CNVs as it typically detects only larger subchromosomal imbalances of ≥7–10Mb at a similar resolution as conventional karyotyping. On the contrary, targeted cfDNA testing on microarray analysis or with SNP‐based testing can screen for a subset of specific pre‐selected individual submicroscopic pCNV with higher sensitivity and specificity than genome‐wide counting assessment. However, a true comprehensive profiling of the fetal genome for microdeletions or microduplications is not possible.^7^

We acknowledge that similar to maternal cfDNA testing, the analysis of cEVTs for aneuploidies has limitations in the setting of feto‐placental mosaicism. However, the analysis of cEVTs has the potential to detect mosaicism (as seen in CVS) by analyzing individual cells. This feature therefore would direct further investigation of these cases and help minimize discordant results with fetal karyotype. Further studies are required to determine the minimum number of cells required to reliably exclude a mosaicism in cEVT analysis and to reliably assess twin zygosity when chorionicity is not informative or when the pregnancy is DC and one fetal genetic profile has been identified. For similar reasoning related to feto‐placental mosaicism, noninvasive cell‐based testing with isolated cEVTs has low clinical utility in pregnancies with a prior high‐risk cfDNA test or the finding of a mosaic trophectoderm biopsy, aneuploidy or large segmental imbalance at PGT‐A, as they rely on the analysis of the same placental layer. While the absence of genomic imbalances on analyzed cEVTs as detected at PGT‐A cannot completely rule‐out the absence of the abnormal cell line in the conceptus, its detection would prompt additional fetal investigations similar to what happens with cytogenetic analysis.

In conclusion, our data support the clinical feasibility of a semi‐automated workflow for a true noninvasive comprehensive fetal genome profiling in order to consistently reduce the residual risk for fetal pathogenic genomic abnormalities down to ∼1Mb in size. These data will be further explored through a larger ongoing validation study in a population enriched for fetal genomic abnormalities.

## CONFLICT OF INTEREST

Anna Doffini, Claudio Forcato, Chiara Mangano, Roberta Aversa, Chiara Maranta, Emilia D. Giovannone, Genny Buson, Chiara Bolognesi, Martina Dori, Liyana Jamal, Raidah B. Ahmad, Thomas J. Musci and Francesca Romana Grati are employees of A. Menarini Biomarkers Singapore Pte Ltd, a Menarini Company. Rebecca Maiocchi, Enrico Ferrazzi and Paola Ricciardi‐Castagnoli are consultant for A. Menarini Biomarkers Singapore Pte Ltd. Between 2020 and 2022, Francesca Romana Grati was an advisory board member and consultant for A. Menarini Biomarkers Singapore Pte Ltd., while she was a full‐time employee of TOMA laboratory. Remaining authors declare no conflicts of interest.

## Supporting information

Supplementary Material

Supplementary Material

Supplementary Material

## Data Availability

Raw data data is not shared. The processed data that support the findings of this study are available from the corresponding author [AD] upon reasonable request.
