# Supplementary material for: Isolation of single circulating trophoblasts from maternal circulation for noninvasive fetal copy number variant profiling
Source: Prenat Diagn. 2022 Dec 8;43(1):14–27. doi: 10.1002/pd.6275 (PMC10107339; doi:10.1002/pd.6275)
Supplement: Supplementary file 1 — Supplementary Material [file PD-43-14-s001.docx]

**Supplementary information**

*Supplementary Tables*

Supplementary Table 1: Description of Coriell cell lines and targets used for determination of CNV resolution. Data were downloaded from the UCSC genome browser (Coriell cell-line CNVs track - <https://genome.ucsc.edu/cgi-bin/hgTrackUi?g=coriellDelDup>).

| Cell line ID | Chromosome location of copy number variation | Start coordinate | End coordinate | Size | Copy number state | ISCN result | Comments reported by Coriell Biobank |
| --- | --- | --- | --- | --- | --- | --- | --- |
| GM09216 | 4 | 144842091 | 144942934 | 100843 | 1 | 46,XY,del(2)(p25.1p23) | CHROMOSOME DELETION |
| GM14164 | 22 | 18876415 | 19025213 | 148798 | 3 | 46,XX,del(13)(q13q32).ish del(13)(q13q32)(RB1-,D13S102+) | TETRALOGY OF FALLOT |
| GM09888 | 22 | 18876415 | 19040071 | 163656 | 3 | 46,XX,del(8)(q23q24.1) | TRICHORHINOPHALANGEAL SYNDROME, TYPE II; TRPS2 (LANGER-GIEDION SYNDROME; LGS) |
| GM11419 | 4 | 144820715 | 145050611 | 229896 | 3 | 49,XYYYY | ANEUPLOID CHROMOSOME NUMBER - NON-TRISOMIC |
| GM07945 | 13 | 20803062 | 21034295 | 231233 | 1 | 46,XY,del(20)(q12q13.1) | ADENOSINE DEAMINASE DEFICIENCY WITH NO IMMUNODEFICIENCY |
| GM16362 | 22 | 22315076 | 22573975 | 258899 | 1 | 47,XY,+del(22)(q11.2q13.3).ish del(22)(q11.2q13.3)(D22Z1+,TUPLE1-,EWSR1-,ARSA+,D22S1726+) | ANEUPLOID CHROMOSOME NUMBER - TRISOMY |
| GM10925 | X | 333274 | 614919 | 281645 | 3 | 46,XY,del(7)(p14p12) | GREIG CEPHALOPOLYSYNDACTYLY SYNDROME; GCPS |
| GM21699 | 3 | 60332 | 606054 | 545722 | 3 | 46,XY,der(6)t(3;6)(p26;q26).ish der(6)t(3;6)(p26;q26)(wcp6+,D62522-,D3S4559+) | CHROMOSOME DELETION |
| GM09888 | 14 | 50822100 | 51655249 | 833149 | 1 | 46,XX,del(8)(q23q24.1) | TRICHORHINOPHALANGEAL SYNDROME, TYPE II; TRPS2 (LANGER-GIEDION SYNDROME; LGS) |
| GM10636 | 2 | 110462483 | 111627422 | 1164939 | 3 | 46,X,dup(X)(p11.3p11.1).ish dup(X)(p11.4p11.1)(STS+,DXZ1++,wcpX+) | DUPLICATED CHROMOSOME |
| GM15603 | 15 | 22280464 | 23487534 | 1207070 | 3 | 46,XY | UNIPARENTAL DISOMY CHROMOSOME 8 |
| GM13464 | 7 | 72725760 | 74142092 | 1416332 | 1 | 46,XY.ish del(7)(q11.23q11.23)(ELN-) | WILLIAMS-BEUREN SYNDROME; WBS |
| GM16362 | 22 | 17467410 | 19077927 | 1610517 | 3 | 47,XY,+del(22)(q11.2q13.3).ish del(22)(q11.2q13.3)(D22Z1+,TUPLE1-,EWSR1-,ARSA+,D22S1726+) | ANEUPLOID CHROMOSOME NUMBER - TRISOMY |
| GM06226 | 1 | 247306531 | 249224376 | 1917845 | 1 | 46,XY,der(1)t(1;16)(q44;p12)mat | TRANSLOCATED CHROMOSOME |
| GM08331 | 21 | 27316123 | 29519188 | 2203065 | 1 | 46,XY,del(13)(q32q33) | CHROMOSOME DELETION |
| GM17942 | 22 | 18650681 | 21462611 | 2811930 | 1 | 46,XY,del(22)(q11.21q11.22).ish del(22)(q11.21q11.22)(TUPLE1-,N85A3+) | DIGEORGE SYNDROME; DGS |
| GM12662 | X | 151909304 | 154929486 | 3020182 | 2 | 46,dup(X)(q28),del(Y)(q11.2).ish del(Y)(q11.2)(DXYS129/DXYS153+,SRY+,DYZ3+,DYZ1+,Z43206+) | CHROMOSOME DELETION |
| GM13476 | 17 | 16763554 | 20395875 | 3632321 | 1 | 46,XX.ish del(17)(p11.2p11.2)(D17S29-) | SMITH-MAGENIS SYNDROME; SMS |
| GM12662 | Y | 24359930 | 28687857 | 4327927 | 0 | 46,dup(X)(q28),del(Y)(q11.2).ish del(Y)(q11.2)(DXYS129/DXYS153+,SRY+,DYZ3+,DYZ1+,Z43206+) | CHROMOSOME DELETION |
| GM22991 | 1 | 752565 | 5310980 | 4558415 | 1 | 46,XX.ish del(1)(p36.32)(CEB108/T7-,SKI-,D1S3739+) | CHROMOSOME 1P36 DELETION SYNDROME |
| GM22624 | 11 | 40476767 | 46074748 | 5597981 | 1 | 46,XX,del(11)(p12p11.2) | POTOCKI-SHAFFER SYNDROME |
| GM21887 | 15 | 22673386 | 28705380 | 6031994 | 1 | 46,XX,del(15)(q11q13).ish del(15)(q11q13)(D15Z1+,SNRPN-,[D15S10/UBE]-,GABRB3-,PML+) | ANGELMAN SYNDROME; AS |
| GM14485 | 8 | 170289 | 7226291 | 7056002 | 1 | 46,XY,der(8)del(8)(p23.1)dup(8)(p23.1p11.2).ish der(8)del(8)(p23.1)dup(8)(p23.1p11.2)(wcp8+,D8S596-) | INVERTED DUPLICATION DELETION |
| GM21699 | 6 | 163662052 | 170982522 | 7320470 | 1 | 46,XY,der(6)t(3;6)(p26;q26).ish der(6)t(3;6)(p26;q26)(wcp6+,D62522-,D3S4559+) | CHROMOSOME DELETION |
| GM14943 | 2 | 235277040 | 243089444 | 7812404 | 1 | 46,XY,del(2)(q37.1).ish del(2)(q37.1q37.3)(D2S447-,D2Z4-) | CHROMOSOME DELETION |
| GM16362 | 22 | 43279613 | 51234443 | 7954830 | 3 | 47,XY,+del(22)(q11.2q13.3).ish del(22)(q11.2q13.3)(D22Z1+,TUPLE1-,EWSR1-,ARSA+,D22S1726+) | ANEUPLOID CHROMOSOME NUMBER - TRISOMY |
| GM21698 | 6 | 162940237 | 170919483 | 7979246 | 1 | 46,XY,del(6)(q26).ish del(6)(q26)(wcp6+,D62522-) | CHROMOSOME DELETION |
| GM10608 | 20 | 9872602 | 18031469 | 8158867 | 1 | 46,XY,del(20)(p12p11.2) | CHROMOSOME DELETION |
| GM10985 | 3 | 60332 | 10330377 | 10270045 | 1 | 46,XX,del(3)(p25) | CHROMOSOME DELETION |
| GM07945 | 20 | 33498254 | 44860471 | 11362217 | 1 | 46,XY,del(20)(q12q13.1) | ADENOSINE DEAMINASE DEFICIENCY WITH NO IMMUNODEFICIENCY |
| GM10989 | 9 | 46586 | 11996831 | 11950245 | 1 | 46,XY,del(9)(p23).ish del(9)(p23)(9ptel30-,D9Z+,wcp9+) | GILLES DE LA TOURETTE SYNDROME; GTS |
| GM08331 | 13 | 98158969 | 110263569 | 12104600 | 1 | 46,XY,del(13)(q32q33) | CHROMOSOME DELETION |
| GM09888 | 8 | 107120037 | 119294603 | 12174566 | 1 | 46,XX,del(8)(q23q24.1) | TRICHORHINOPHALANGEAL SYNDROME, TYPE II; TRPS2 (LANGER-GIEDION SYNDROME; LGS) |
| GM20556 | 15 | 20016315 | 32770448 | 12754133 | 4 | 47,XY,+idic(15)(q13).ish idic(15)(q13)(D15Z1++,D15S11++,GABRB3++) | ISODICENTRIC CHROMOSOME |
| GM06936 | 10 | 104426 | 12878926 | 12774500 | 1 | 46,XX,del(10)(p13) | CHROMOSOME DELETION |
| GM09102 | 11 | 120490978 | 134944770 | 14453792 | 1 | 46,XY,del(11)(q23.3) | CHROMOSOME DELETION |
| GM06870 | 18 | 11542 | 15401751 | 15390209 | 4 | 47,XX,+i(18)(p10) | ANEUPLOID CHROMOSOME NUMBER - NON-TRISOMIC |
| GM16595 | 5 | 8633804 | 24036642 | 15402838 | 1 | 46,XX,del(5)(p15.2p14).ish del(5)(p15.2p14)(C84C11T7+,D5S721-,D5S23-,EGR1+) | CRI-DU-CHAT SYNDROME |
| GM10925 | 7 | 38632016 | 54714504 | 16082488 | 1 | 46,XY,del(7)(p14p12) | GREIG CEPHALOPOLYSYNDACTYLY SYNDROME; GCPS |
| GM09216 | 2 | 10343536 | 27151878 | 16808342 | 1 | 46,XY,del(2)(p25.1p23) | CHROMOSOME DELETION |
| GM11213 | 2 | 187110202 | 204602929 | 17492727 | 1 | 46,XX,del(2)(q32.1q33) | CHROMOSOME DELETION |
| GM10636 | X | 39808590 | 57956790 | 18148200 | 3 | 46,X,dup(X)(p11.3p11.1).ish dup(X)(p11.4p11.1)(STS+,DXZ1++,wcpX+) | DUPLICATED CHROMOSOME |
| GM05966 | 14 | 54968765 | 76147660 | 21178895 | 3 | 46,XY,dup(14)(q22q24) | DERIVATIVE CHROMOSOME |
| GM06226 | 16 | 85814 | 21389970 | 21304156 | 3 | 46,XY,der(1)t(1;16)(q44;p12)mat | TRANSLOCATED CHROMOSOME |
| GM10800 | 4 | 70061848 | 95078093 | 25016245 | 1 | 46,XY,del(4)(q13.2q22) | CHROMOSOME DELETION |
| GM22601 | 4 | 65664 | 25981953 | 25916289 | 1 | 46,XY,del(4)(p15.2) | WOLF-HIRSCHHORN SYNDROME; WHS |
| GM11419 | Y | 2650425 | 28799923 | 26149498 | 4 | 49,XYYYY | ANEUPLOID CHROMOSOME NUMBER - NON-TRISOMIC |
| GM11672 | 10 | 49292450 | 75450707 | 26158257 | 1 | 46,XY,del(10)(q11.2q22.1) | CHROMOSOME DELETION |
| GM14485 | 8 | 12528415 | 43600368 | 31071953 | 3 | 46,XY,der(8)del(8)(p23.1)dup(8)(p23.1p11.2).ish der(8)del(8)(p23.1)dup(8)(p23.1p11.2)(wcp8+,D8S596-) | INVERTED DUPLICATION DELETION |
| GM09367 | 6 | 107754362 | 143064154 | 35309792 | 3 | 46,XX,dup(6)(q21q24).ish dup(6)(q21q24)(wcp6+) | DUPLICATED CHROMOSOME |
| GM05067 | 9 | 46586 | 39778619 | 39732033 | 3 | 47,XY,+del(9)(q11)mat | ANEUPLOID CHROMOSOME NUMBER - TRISOMY 9 |
| GM12606 | 13 | 19045627 | 60241421 | 41195794 | 3 | 47,XY,+del(13)(q21.2) | CHROMOSOME DELETION |
| GM14164 | 13 | 47802083 | 95714976 | 47912893 | 1 | 46,XX,del(13)(q13q32).ish del(13)(q13q32)(RB1-,D13S102+) | TETRALOGY OF FALLOT |
| GM13019 | X | 168464 | 56895584 | 56727120 | 1 | 46,X,idic(X)(p10)[25]/46,X,del(X)(p10)[16]/45,X[9] | TURNER SYNDROME |
| GM20022 | 3 | 134562094 | 195652973 | 61090879 | 3 | 46,XY,dup(3)(q21q29).ish dup(3)(q21q29)(wcp3+,D3S4560+) | DUPLICATED CHROMOSOME |
| GM17867 | X | 168464 | 154929486 | 154761022 | 2 | 47,XXY | XXY SYNDROME; KLINEFELTER SYNDROME |
| GM01416 | X | 168464 | 155182342 | 155013878 | 4 | 48,XXXX | XXXX SYNDROME |
| GM20027 | X | 168464 | 155233846 | 155065382 | 1 | 45,X | TURNER SYNDROME |

**Supplementary table 2**: cEVTs distribution by profile quality.

| **Profile quality** | **cEVTs** | **%** | **Used for evaluation** |
| --- | --- | --- | --- |
| ***High-quality*** | 378 | 87.5 | yes |
| ***S-phase*** | 34 | 7.9 | partially* |
| ***Apoptotic*** | 13 | 3.0 | no |
| ***Low-quality*** | 7 | 1.6 | no |
| ***Total*** | 432 |  |  |

* 25/34 S-phase cells show copy-number profiles with enough quality to be used for aneuploidy detection.

Supplementary table 3: Aneuploidy results obtained by analysis of abnormal cEVTs. All cases with abnormal fetal karyotype were correctly identified; in one case with normal karyotype (M140) a confined placental mosaicism (CPM) for T16 was detected in cEVTs.

| **Patient ID** | **Indication for invasive PDx** | **cEVTs analysis** | | | **Invasive prenatal diagnosis** | |
| --- | --- | --- | --- | --- | --- | --- |
|  |  | **N° cEVTs** | **Autosomes** | **Sex**  **chromosomes** | **Karyotype** | **Prenatal tissue analyzed** |
| M015 | Positive FCT - high NT | 6 | T21 | XY | 47,XY,+21 | CVSc |
| M019 | Positive FCT - high NT | 6 | T21 | XX | 47,XX,+21 | CVSc |
| M038 | Positive cfDNA for T21 | 1 | T21 | XY | 47,XY,+21 | CVSc+CVSm |
| M057 | Positive FCT - high NT | 2 | T18 | XY | 47,XY,+18 | CVSc |
| M081 | Positive FCT - high NT | 1 | T21 | XY | 47,XY,+21 | CVSc+CVSm |
| M085 | Positive FCT - blood test | 2 | T21 | XX | 47,XX,+21 | CVSc+CVSm |
| M094 | Ultrasound scan anomalies | 2 | T21 | XX | 47,XX,+21 | CVSc+CVSm |
| M096 | Positive FCT - high NT | 25 | 66 | XXY | 69,XXY | CVSc+CVSm |
| M109 | Positive FCT - high NT | 1 | T21 | XY | 47,XY,+21 | CVSc |
| M115 | Positive cfDNA for T21 | 3 | T21, mosaic T18 | XY | 47,XY,+21 (cCVS)  48,XY,+18,+21 (mCVS) | CVSc+CVSm |
| M116 | Positive FCT - high NT | 2 | T21 | XY | 47,XY,+21 | CVSc+CVSm |
| M124 | Ultrasound scan anomalies | 2 | T21 | XY | 47,XY,+21 | CVSc |
| M140 | Positive cfDNA for T16 | 3 | T16 | XX | 46,XX | AF |
| M141 | Positive FCT - high NT | 1 | Euploid | Monosomy X | 45,X | CVSc |
| M191 | Positive FCT - blood test | 6 | T21 | XY | 92,XXYY,der(14;21), +21,+21 (cCVS)  46,XY,der(14;21),  +21 (mCVS) | CVSc+CVSm |
| M202 | Positive FCT - blood test | 4 | T21 | XX | 47,XX,+21 | CVSc+CVSm |
| M208 | Positive FCT - high NT | 2 | T21 | XX | 47,XX,+21 | CVSc+CVSm |

Abbreviations: cEVTs, circulating Extravillous Trophoblasts; FCT, First trimester Combined Test; NT, nuchal translucency; cfDNA, cell-free DNA; T21, trisomy 21; T18, trisomy 18; T16, trisomy 16; CVSc, analysis of cytotrophoblast layer by direct method; CVSm, analysis of mesenchyme by long-term culture; AF, amniotic fluid.

**Supplementary methods**

Copy-number calling

Copy-number profiles were obtained using an in-house developed pipeline based on the common approach for copy-number aberration (CNA) detection in single-cell sequencing^1^. Briefly, it includes a first step of alignment of DNA sequences to the reference genome (hg19). Then, alignments were sorted and filtered for mapping quality and read counts were obtained for non-overlapping genomic bins, except for low mappability regions. Counts were normalized accounting for *Ampli*1 WGA specific bias, converted in bin ratios, then segmented and rounded to the nearest integer. Significance of each copy-number call was assessed by performing Wilcoxon rank-sum test and then adjusted using Benjamini–Hochberg correction for multiple tests. Sample sex was automatically determined based on the fraction of total reads mapped on chromosome Y.
A bin size of 500Kb and 100Kb was used for aneuploidy and CNV detection, respectively. The analysis involved only cells with a suitable copy-number profile, characterized by low background noise and the absence of variable aberrations across the genome (chaotic profiles). The evaluation of profile quality was done using hard cut-offs for noise metrics, such as DLRS<0.35. Moreover, each single copy-number profile was manually inspected and reviewed to get a final classification.

Determination of CNV size resolution

Experiments with cell lines and single cells were aimed to determine the analytical sensitivity of our method. Specifically, each target of the Coriell panel was compared with the CNVs called by our pipeline for each sample (gDNA aliquot or single cell) obtained by the different cell lines. FASTQ files downsampled to 3M reads were processed with our in-house developed copy-number calling pipeline using a bin size of 100Kb. Profile quality parameters, such as DLRS (>0.35), together with visual inspection were used to exclude low-quality samples. CNV target coordinates and expected copy-number levels were downloaded from the UCSC genome browser (<https://genome.ucsc.edu/cgi-bin/hgTrackUi?g=coriellDelDup>)^2^. For each target, the analytical true positives (TP), true negatives (TN), false positives (FP) and false negatives (FN) were determined by evaluating the expected copy-number against the segmented signal obtained for a specific sample in that region, imposing a minimum overlap >35% between target and copy-number segment and a minimum fraction of copy-number signal in the considered target. This was necessary since low-mappability or highly-repetitive regions are masked in our pipeline and copy-number signals are not generated in the corresponding genomic bins. If the bin fraction with copy-number signal does not exceed 35%, the target was considered "no call". Targets were defined as “no eval” if the cell-line, from which the sample originated, harbored an overlapping alteration target.

**Supplementary Figures**

**Supplementary Figure 1:** Whole-genome False Positive (FP) evaluation.

Distribution of analytical false positive CNVs according to their length for the analyzed Coriell gDNAs and single cells. False positives were defined as the alterations detected by our pipeline along the entire genome (not in specific targets) but not confirmed by Coriell expected targets. A) The density plot clearly shows that the majority of the few false positives has a size lower than 1Mb for both gDNA and single cells. B) Percentage of samples with increasing numbers of False Positives. The great majority of gDNA (>95%) and single cell (>80%) samples show one FP at maximum.

**Supplementary Figure 2:** Copy-number profiles of Coriell cells.

Examples of expected CNVs identified from Coriell cell lines in the single cell experiment. Highlighted in green are the expected alterations according to UCSC Coriell CNV track.

A) 1.6 Mb duplication on chr22 for GM16362. Although the size of this CNV is above our limit of detection, its location across the pericentromeric region of chr22 makes the identification of this imbalance more complicated (approximately in 50% of cells) and shorter than expected (~700Kb).

B) 800 Kb deletion on chr14 for GM09888. This is the smallest CNV reliably identified; the actual size of the segments identified with the copy-number caller algorithm spans from 0.9 Mb to 1.1 Mb.

C) 2.8 Mb deletion on chr22 for GM17942 (DiGeorge Syndrome). Although located in a region characterized by low complexity, the deletion responsible for the DiGeorge Syndrome is reliably identified in all cases.

**References**

1. Mallory XF, Edrisi M, Navin N, Nakhleh L. Methods for copy number aberration detection from single-cell DNA-sequencing data. *Genome Biol.* 2020; **21**(1):208
2. Tang Z, Berlin DS, Toji L, *et al.* A dynamic database of microarray-characterized cell lines with various cytogenetic and genomic backgrounds. *G3: Genes, Genomes, Genetics* 2013; ***3***(7):1143–1149
