# Supplementary figures and images for: Isolation of single circulating trophoblasts from maternal circulation for noninvasive fetal copy number variant profiling
Source: Prenat Diagn. 2022 Dec 8;43(1):14–27. doi: 10.1002/pd.6275 (PMC10107339; doi:10.1002/pd.6275)

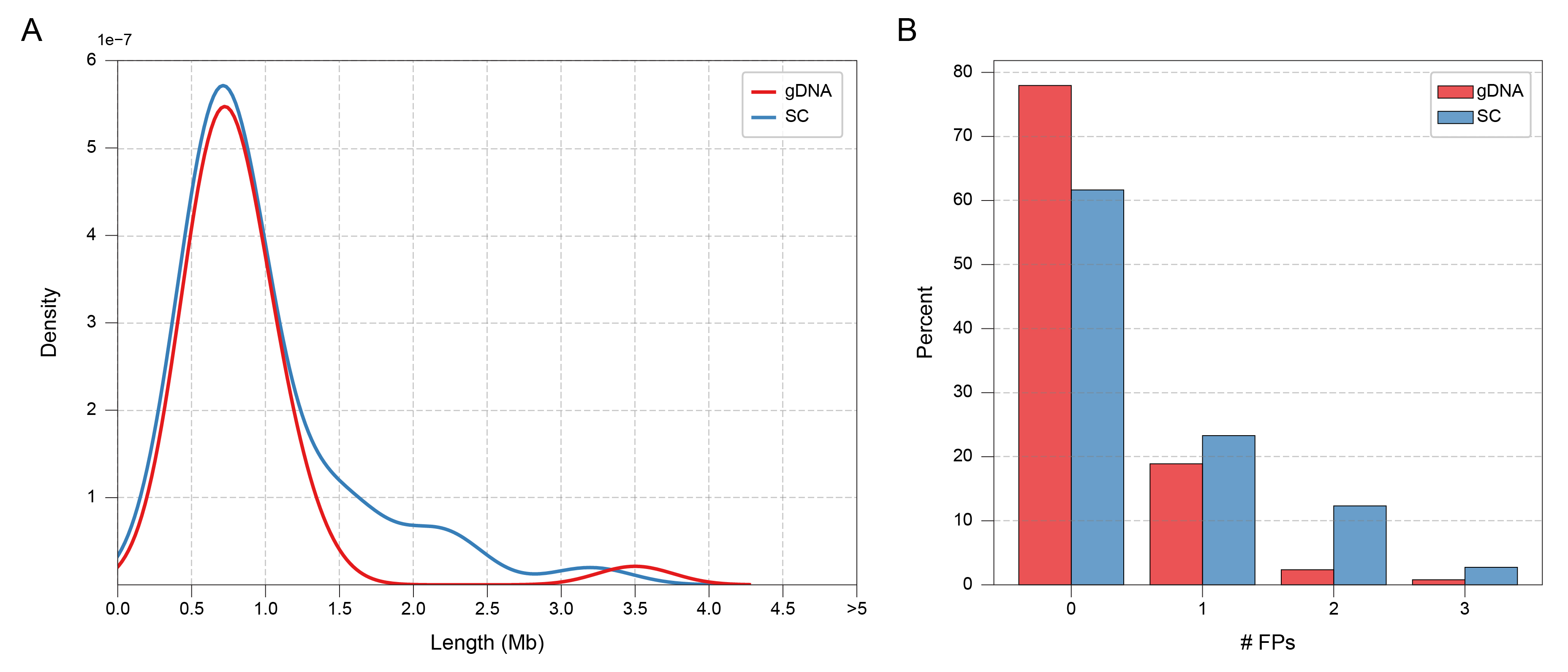

Supplement: Supplementary file 2 — Supplementary Material [file PD-43-14-s003.png]

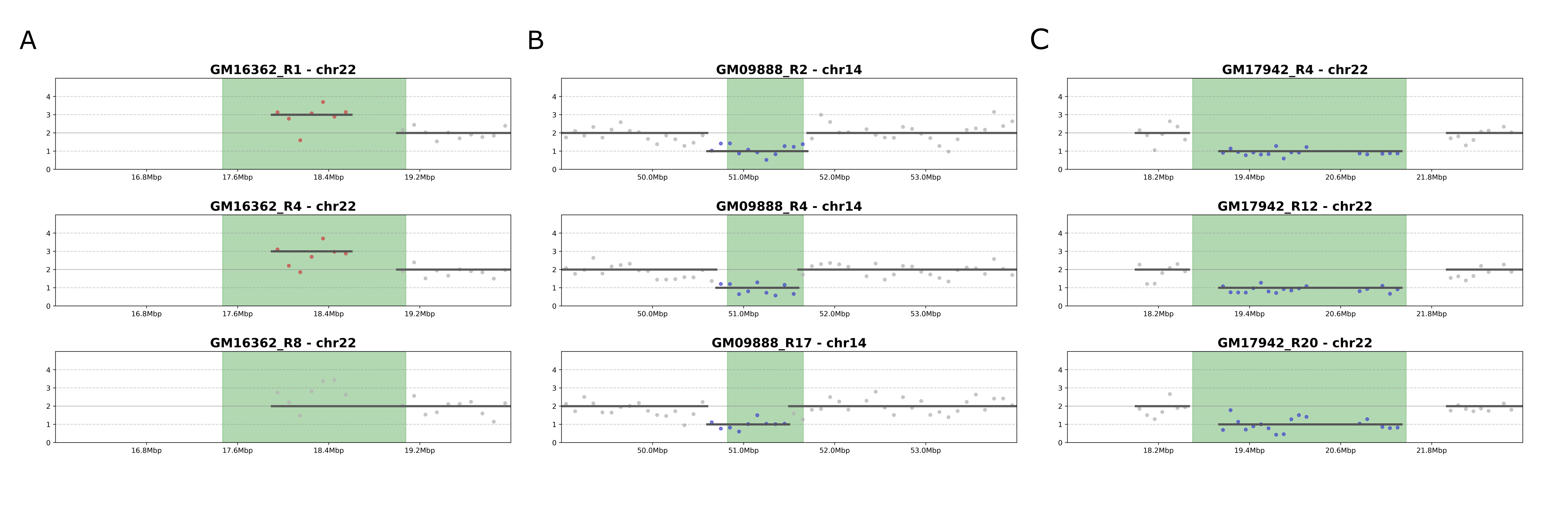

Supplement: Supplementary file 3 — Supplementary Material [file PD-43-14-s002.png]
